# Development and validation of a questionnaire to assess delay in treatment for breast cancer

**DOI:** 10.1186/1471-2407-12-626

**Published:** 2012-12-28

**Authors:** Karla Unger-Saldaña, Ingris Peláez-Ballestas, Claudia Infante-Castañeda

**Affiliations:** 1 Faculty of Medicine, Universidad Nacional Autónoma de México & Instituto Nacional de Cancerología de México, Mexico City, Mexico; 2 Rheumatology Department, Hospital General de México, Mexico City, Mexico; 3 Faculty of Medicine, Universidad Nacional Autónoma de México, Mexico City, Mexico

**Keywords:** Breast cancer, Delayed medical care, Questionnaire, Validity, Reliability

## Abstract

**Background:**

This study reports the reliability and validity of a questionnaire designed to measure the time from detection of a breast cancer to arrival at a cancer hospital, as well as the factors that are associated with delay.

**Methods:**

The proposed questionnaire measures dates for estimation of the patient, provider and total intervals from detection to treatment, as well as factors that could be related to delays: means of problem identification (self-discovery or screening), the patients’ initial interpretations of symptoms, patients’ perceptions of delay, reasons for delay in initial seeking of medical care, barriers perceived to have caused provider delay, prior utilisation of health services, use of alternative medicine, cancer-screening knowledge and practices, and aspects of the social network of support for medical attention. The questionnaire was assembled with consideration for previous research results from a review of the literature and qualitative interviews of patients with breast cancer symptoms. It was tested for face validity, content validity, reliability, internal consistency, convergent and divergent validity, sensitivity and specificity in a series of 4 tests with 602 patients.

**Results:**

The instrument showed good face and content validity. It allowed discrimination of patients with different types and degrees of delay, had quite good reliability for the time intervals (with no significant mean differences between the two measurements), and fairly good internal consistency of the item dimensions (with Cronbach’s alpha values for each dimension between 0.42 and 0.85). Finally, sensitivity and specificity were 74.68% and 48.81%, respectively.

**Conclusions:**

To the best of our knowledge, this is the first published report of the development and validation of a questionnaire for estimation of breast cancer delay and its correlated factors. It is a valid, reliable and sensitive instrument.

## Background

Breast cancer is the most common cancer in women and the main cause of cancer-related deaths worldwide, causing approximately 2-million new cases and 500,000 deaths in 2008 [1]. It is also the main cause of cancer-related deaths among women in Mexico, with close to 14,000 new cases and 5,000 deaths per year [1].

In Mexico, as in other developing countries, breast cancer survival rates are much lower than in developed countries, mainly because cancer is diagnosed in later stages. For instance, in the United States, 60% of breast cancer cases are diagnosed in stages 0 and I, with survival rates of 98% [2], whereas in Mexico less than 10% of patients are diagnosed in these early stages and 47% in the most advanced stages (III and IV) [3,4]. The main reasons for presentation of breast cancer patients in advanced stages in Mexico could be related to the very low participation of women in breast cancer screening tests [5], delayed help-seeking for breast cancer symptoms and barriers to accessing health care services [6].

Breast cancer total delay is defined in the literature as a span of more than three months between the discovery of symptoms by the patient and the beginning of definitive cancer treatment [7-9]. Traditionally, it has been classified in two types: *patient* and *provider delay.* Cut-points to define these intervals vary across studies, but the majority of studies have considered patient delay to be more than three months between the discovery of symptoms and the first medical consultation [7,10-12]. In turn, provider delay takes place between the first medical consultation and the beginning of definitive treatment, and the most accepted threshold is one month, although this cut-point varies across studies [13-17].

Although breast cancer treatment delay has been studied by multiple authors for years, there is no validated instrument to measure time intervals and correlated factors. Several instruments to estimate the likelihood of delay in seeking medical attention if a cancer symptom were to present have been validated in asymptomatic patients [18,19], but no studies have assessed delay among breast cancer patients. Some breast cancer delay studies refer to questionnaires based on previous study results or describe the results of pilot studies, but no validity and reliability measures are specified [20-22]. We found only one study in which the reliability and validity of self-reported symptoms and dates of diagnostic tests was measured, but this study surveyed patients with colon cancer [23], which is characterised by a completely different set of symptoms, natural history and illness behaviour compared to breast cancer [24-27]. Nonetheless, the authors found that self-reported symptoms, tests and dates were in general reliable but not necessarily valid [23].

This dearth of validated instruments that measure delay is most likely a result of the difficulties inherent in establishing the validity of time intervals for medical care for any disease [28]. These intervals are estimated based on the date of symptom discovery, the date of initial medical consultation and the date of beginning of treatment. The most problematic dates to obtain reliably are those given by the patient, as there may be recall inaccuracy [29] and response bias due to concerns about social desirability [30]. Nevertheless, it has been shown that patients commonly recall the precise time when they first discovered their symptoms [12,23,31].

Due to the known association between delay, advanced clinical stages of breast cancer and survival [32], it is important to quantify the degree of delay and its correlated factors so that targeted interventions to reduce delay can be designed. Such interventions are especially important in developing countries where the majority of patients are diagnosed in advanced stages, as in Mexico [33].

In this paper, we report the development and validation of a questionnaire designed to a) measure the *time from detection of a possible breast cancer* (through either patient symptoms or abnormal screening findings) to arrival at a cancer hospital and b) identify factors correlated with the delayed beginning of medical treatment.

## Methods

### Study design

**Figure 1:**
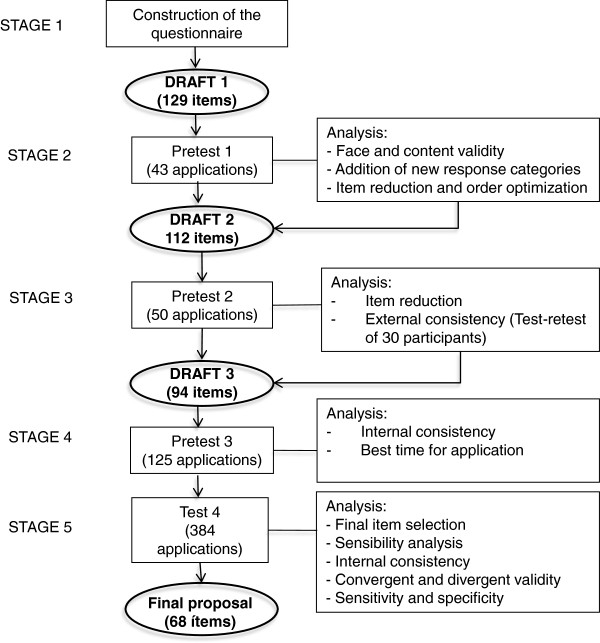
**Stages in the production of the breast cancer delay questionnaire.** This schema summarizes the stages of construction and validation of the questionnaire.

The study protocol was approved by the scientific and ethics review boards of the Mexican National University (registry number 24–2007) and the National Cancer Institute (registry 05048TMI). The results here presented are part of a larger study that aims to quantify time intervals from the detection of a possible breast cancer to the beginning of cancer treatment and identify the main factors predicting prolongation of these intervals. This paper reports the development and validation (using standard test-construction methods) of an instrument to assess time from detection of breast cancer to arrival at a cancer hospital and possible associated factors with delayed beginning of treatment [28,34]. After the first draft of the questionnaire was written, it was tested for psychometric properties and refined in a set of 4 cross-sectional tests of different patients. The construction and validation of the questionnaire are schematised in Figure 1.

### Construction of the questionnaire

The questionnaire was built to assess time from the first identification of a breast problem that could be cancer (either through screening practices or symptoms discovered by the patient) to arrival at a cancer hospital, and possible associated factors with delayed beginning of treatment. The questionnaire was assembled with consideration for previous research results from a review of the literature and qualitative interviews of patients with breast cancer symptoms. Details of these previous study phases are published elsewhere [9,35]. Nevertheless, it is important to emphasise that the definitions and classifications of the time intervals, as well as those of the correlated factors and the methods used for their study, were critically assessed and considered in the construction of the instrument.

Furthermore, the first draft of the questionnaire included items reflecting the most common themes in the qualitative patient interviews. The instruments’ questions and response categories were phrased in simple terms and in accordance with the words used by the patients in the qualitative interviews. The items were then revised and improved after evaluation by experts in the design of questionnaires and a multidisciplinary team of advisors: a sociologist, a clinical epidemiologist and anthropologist, an oncologist, an epidemiologist and a statistician. Finally, they were tested for comprehensibility by the participants in the first pilot study.

The questionnaire was designed for application through face-to-face interviews due to the difficulties associated with self-administered research instruments in Mexico among people with little formal education [36,37], like our study participants.

The conceptual and operational definitions of the time intervals agree with the majority of studies of breast cancer delay and recent recommendations of a consensus [38]. *Total interval* was defined as the time from identification of the problem (either through symptoms or screening) to the beginning of cancer treatment; *patient interval* was defined as the time from identification of the problem to the first medical consultation; and *provider interval* was defined as the time from the first presentation (first medical consultation) to the beginning of cancer treatment. *Date of symptom discovery* and *date of presentation* were both obtained from the patients through the questionnaire, whereas the *dates of beginning of treatment* were obtained from their hospital charts.

### Participants

The patients included in this study were women who were referred to the Breast Tumours Department of the Mexican National Cancer Institute (INCAN) with a probable breast cancer diagnosis. INCAN is a referral hospital that offers specialised cancer care at low costs for uninsured patients, most of whom are unemployed or informally employed.

Patients were excluded if they had received previous cancer treatment for the current breast cancer, if they had a personal history of cancer, if they were unable to participate in the interview (because of impaired hearing, inability to communicate in Spanish, or mental disability), or if they had a history of a benign breast condition that had been under medical surveillance. This last exclusion criterion was considered because the profile of women with benign breast disease has been shown to differ significantly from that of women with breast cancer [9]. Additionally, completed questionnaires were excluded from analysis if the patient did not recall one or more of the relevant dates.

### Interviews

In the first pilot study, KUS conducted all of the interviews. From the second pilot study onwards, the interviewers were psychologists who were trained by KUS to standardise the interviewing process. Training consisted in a first theoretical module, a second phase of supervised practice of interviews with individuals that simulated fictitious patient stories, and a third phase where the interviewers administered the questionnaire to breast cancer patients in the presence of KUS. The patients who were interviewed in the training stage were not included in the final analysis.

New patients who arrived at the Breast Tumours Department were invited to participate while they were waiting for their first consultation with the breast specialist. The interviewer identified herself and asked the patient for verbal consent to ask her three questions (the exclusion criteria). If the patient was eligible for the study, she was then invited into a private room with the person who accompanied her to the hospital. No monetary incentives were given. The patient was given a written description of the study objectives and what her participation would consist of, including her rights to not participate. The trained interviewer read this form with the patient and relative and then offered to clarify any issues that remained ambiguous. Finally, if she was willing to participate, she was asked to sign an informed consent form, and her relative was asked to sign as a witness. After informed consent was obtained, the patient’s relative was asked to wait for the patient outside so that the interview could be done in private.

All dates were retrieved in the form of day, month and year with the aid of calendars and in relation to events that occurred during the patient’s help-seeking trajectory, including national holidays, significant news and personal events such as the participant’s birthday. Calendars were printed out, and as part of the standardised interview process, the interviewers gave each participant a calendar and asked her to recall the relevant dates as precisely as she could. This procedure enhanced recall, as the participant could deduce the date of a health event from the calendar by locating it in relation to other significant dates.

For participants who had lesions detected by screening, the date of problem identification was considered to be the date of first contact with a medical service; thus, the patient interval was 0 days. When the patient did not remember the exact date, she was asked to remember whether it was in the beginning, middle or end of the month. The beginning of the month was coded as day 5, the middle of the month as day 15, and the end of the month as day 25. If she could not be more precise, her answer was coded as day 15 (middle of the month). Some patients with several years of delay could remember only the year. These patients were asked to remember if the date was early in the year, mid-year or late in the year. These answers were coded as follows: “beginning of the year”, 15 February; “middle of the year”, 15 June; and “end of the year”, 15 November. If she remembered only the year, the answer was coded as “middle of the year”.

Participants were not directed in any way by the interviewers to obtain an answer. Part of the standardisation consisted in reading each question to the patient two times word-for-word if she did not understand the first time. If the participant still did not understand after the second time the question was asked, then the interviewers paraphrased the question. Finally, if no response was elicited after the third attempt, the interviewers marked that question as unanswered and went on to ask the next item.

### Review of patient files

To quantify the total delay and provider intervals, the dates required are that of problem identification, first medical consultation and beginning of treatment. The instrument was designed to assess only the first two, since the patients were interviewed before they began cancer treatment. Therefore, the participants’ medical files were reviewed six months after the interview to obtain the date that the first cancer treatment was begun and the clinical stage of the cancer. The beginning of cancer treatment was considered as the date that the first oncologic treatment was begun, whether it was surgery, chemotherapy, radiotherapy, hormonal therapy or antibody therapy.

### Procedures and measures

#### Sensibility analysis

Sensibility was appraised in a qualitative manner by the researchers in terms of what the instrument contains and what it does, as recommended by Feinstein [28]. The following topics were analysed: a) comprehensibility, b) replicability, c) suitability of scale, d) face validity, e) content validity, f) ease of usage and g) patient acceptance. This assessment was performed continuously throughout the different stages of the study. Items that were irrelevant or duplicated in early versions of the questionnaire were eliminated. Those that were found by respondents to be ambiguous or difficult to understand were rewritten, and some new categories were incorporated when an answer that had not been considered in the previous version was given by more than 10% of the interviewees. Finally, the item order was changed after the first and second pre-test studies to make the interview more fluent.

#### Reliability

External consistency or reliability was estimated with a test-retest analysis of 30 patients who participated in the second pre-test and to whom the same instrument was applied twice within a 3-month interval. Appointments for the second interview with these participants were scheduled by telephone. The second interview was arranged at a date and time that was convenient for the patient and on the same day as one of her medical appointments so that she did not have to come to the hospital only for the interview.

#### Best timing for questionnaire administration

Different timings for the interviews were tested to maximise patient cooperation and minimise interference with the usual institutional procedures. This was conducted in a qualitative manner by the researchers. Furthermore, the proportion of patients that reported dates with precision was compared for the participants in the test-retest.

#### Validity

Criterion validity was not assessed, as there is no gold-standard instrument to measure delay or the factors associated with it. Construct validity was evaluated for each questionnaire dimension by estimating internal consistency and convergent and divergent validity [28,39].

#### Item scoring

Responses within each item were assigned a value according to their bivariate association with the different time intervals (total, patient and provider). More points were given to the response categories associated with longer intervals, and fewer points were given to those associated with shorter intervals. For each item the response category associated negatively with delay was given a value of 0, the category with the highest positive association with delay was given the highest value, and the remaining response categories were given values in between accordingly. The assigned value for each item’s response categories depends on the number of response categories. If there were four response categories, the lowest value would be 0 and the highest 3, whereas if there were two categories the values would range only from 0 to 1. For example, the item “means of problem identification” had a score of 0 when the problem was identified through screening and 1 when it was identified through patient symptoms. To determine a score for each dimension of the questionnaire, the component item scores for each patient were summed. Finally, to determine the total questionnaire score, scores for all dimensions were added up.

#### Sensitivity and specificity

ROC curves were estimated for the total questionnaire scores in relation to total, patient and provider delays. As there is no gold standard, the total questionnaire score was dichotomised using the median as the cut-off point [28,39]. The intervals were also dichotomised with the following cut-off values: total delay was recorded when the total interval was equal to or greater than 180 days; and patient or provider delay was recorded when the patient or provider interval was 90 days or more. We decided to use cut-off points that are much longer than the most widely accepted thresholds in the literature because 97% of the women in our sample had total intervals greater than 90 days.

#### Final item selection

Bivariate analysis of each of the questionnaire items and the three time intervals was performed to identify the factors most likely to predict delays. Those items that showed the highest correlations were kept for the final version of the questionnaire. Those that were not statistically significant but hold theoretical relevance were also kept in the final version of the questionnaire. The purpose of this study was to assess the instruments’ validity and reliability, not draw conclusions regarding the relevance of each item to delay. In the next phase of the study, a multivariate analysis of a larger, multi-centre sample will allow determination of each item’s relevance and further refinement of the instrument.

#### Statistical analysis

Sample sizes were based on the quality criteria recommended by Terwee et al., who suggested a minimum of 50 patients for assessing construct validity and a minimum of 100 patients for assessing internal consistency [40].

Descriptive statistics were estimated to measure the participants’ sociodemographic characteristics, final diagnoses and clinical stages, as well as for the total, patient and provider intervals. The quantitative variables included mean, median, standard deviation and interquartile range; categorical variables included frequency and percentage.

To reduce the questionnaire, a correlation matrix of variables and a principal component analysis of each dimension were carried out. For pairs of variables with correlation coefficients higher than 0.70, we selected the one that explained the greatest variation according to the principal component analysis.

To evaluate test-retest reliability, a *t*-test for related samples was performed to compare mean differences in interval lengths between the two measurements. A correlation matrix was estimated for the remaining variables to compare responses between the first and second questionnaire applications for each individual. Furthermore, the total score obtained by each patient on the first and second application of the questionnaire was compared for each dimension.

Internal consistency was assessed through estimation of Cronbach’s alphas for each questionnaire dimension, as recommended by Terwee and collaborators [40]. Alphas equal to or greater than 0.40 (p < 0.05) were considered acceptable [34,41]. Convergent validity was assessed by estimating Spearman correlation coefficients for items comprising each domain, and divergent validity across items of different domains.

To select the final items in the instrument, bivariate associations were estimated between each questionnaire item and each of the three intervals (total, patient and provider) through Likelihood Ratio (LR) coefficients and bivariate logistic regression. Items that showed LR coefficients greater than 10 (p < 0.05) and significant ORs (p < 0.05) were considered to have statistical relevance.

## Results

### Description of the instrument: the breast cancer delay questionnaire

The final questionnaire is composed of two independent modules containing 68 items. These items mainly include categorical answers, Likert scales and several open-ended questions. One module assesses dates to estimate the intervals in which delay can occur. The other measures different factors predisposing to delay. The two modules are intertwined in the questionnaire to facilitate its application. In the first pilot study, we tested the two modules separately and found that it was more difficult to elicit responses. On the one hand, it was harder for the patient to recall the dates of symptom discovery and first medical consultation without having discussed how they discovered the first symptom or where they sought medical attention for their health problem. On the other hand, questions about reasons for having delayed care or barriers to accessing care required respondents to think about dates and how much time had passed.

The combined application of this instrument and a revision of patients’ clinical files to assess the dates of diagnosis confirmation and the beginning of cancer treatment allow the estimation of total, patient and provider intervals, as well as of the subintervals that compose the provider intervals: diagnosis and treatment intervals. The instrument is organised in 8 questionnaire sections that encompass 16 dimensions. Table 1 shows the questionnaire dimensions and the included items. The questionnaire in Spanish, is available for free in the BMC Cancer webpage (Additional file 1). Although the attached version of the questionnaire does not include sociodemographic items, it is certainly recommended that these are added in accordance to the context where it will be administered.

**Table 1:** Questionnaire dimensions and items

**1. Means of problem identification** (self discovery or screening)	
**2. First symptom identified by the patient**	
**3. Patient initial interpretations of symptoms**	
Perceived seriousness	Initial worry
Initial interpretation of cancer	
**4. Breast cancer symptoms present at arrival to the cancer hospital**	
Lymphadenopathies	Breast ulcer
Breast pain	Breast pruritus
Arm pain	Breast enlargement
Paresthesias of breast and/or ipsilateral arm	Form changes of the breast
Breast skin changes	Nipple discharge
**5. Most worrisome symptom for the patient**	
**6. Patient's reason for seeking medical care** (appearance of symptoms/persistence of symptoms/worsening of symptoms)	
**7. Patient's perception of delay**	
Perception of patient delay	Perception of provider delay
**8. Patient's perceived reasons for patient delay**	
Thought symptoms would resolve alone	Carelessness/neglect
Didn't know where to seek care	Fear
Lack of financial resources	Embarrassment
Difficulty to miss work	Taking care of young children, older or ill relatives
**9. Patient's perceived barriers to have caused provider delay**	
Lack of information of health services	Lack of financial resources
Fear	Difficulty to miss work
Perceived errors in diagnosis of first doctors consulted	Perceived long waiting times for medical appointments
Had to take care of youngsters, elders or ill relatives	Had to borrow money to get medical care
Financing source for payment of health care	
**10. Health service utilization**	
First health service used (public, private, other)	Breast ultrasound requested by first doctor consulted
Number of different health services consulted	Biopsy requested by first consulted doctor
Diagnostic impression of the first doctor consulted	Health service of referral to hospital
Mammogram requested by the first consulted doctor	Biopsy done previous to arrival at hospital
**11. Social network support for seeking medical attention**	
Instrumental support	Sex of person of most support
Emotional support	Size of network activated for breast problem
Recommendations to consult a doctor	Sex of social network members
Financial support	Kinship with members of her social network
Company to medical consultations	Sex of first person who she told
Kinship with person of most support	Number of women older than 15 living at same household
**12. Time between identification of the problem and the first time to talk to someone about it**	
**13. Use of alternative medicine**	
**14. Cancer-related knowledge**	
Knowledge of a person with cancer	Has heard about mammograms
Knowledge of mammograms' utility	Knowledge of recommended age for first screening mammogram
**15. Cancer screening practices**	
Last Pap smear	Screening Breast Clinical Exam before current breast problem
Mammograms before current breast problem	Breast Self Examination practice
**16. Dates**	
Symptom discovery	First medical consultation

### Pre-test 1. Face and content validity (Stage 2)

The first version of the questionnaire, which comprised 129 items, was applied to 43 patients who came to the Breast Clinic of INCAN for the first time between January and May 2008 with a probable breast cancer diagnosis (Figure 1). Patient demographic, disease and delay data are summarised in Table 2. The aim of this stage was to evaluate the instrument’s sensibility [28]. The most important modifications to the questionnaire that arose from this stage were 1) rearrangement of the sequence of items to make the interview more fluent, 2) rewording of questions, 3) addition of response categories, and 4) elimination of items that showed no variation.

**Table 2:** **Demographic and disease information**^**a**^

	***Pretest 1***	***Pretest 2***	***Pretest 3***	***Test***
	***(n=43)***	***(n=50)***	***(n=125)***	***(n=384)***
***Age (years)***				
Mean (SD, range)	47.51 (12.29, 27–78)	51.08 (11.96, 28–81)	50.96 (12.12, 28–86)	49.61 (12.85, 18–91)
***Marital status***				
Married or living together	23 (54)	33 (66)	69 (55)	216 (56)
Other	20 (46)	17 (34)	56 (45)	168 (44)
***School education***				
Analphabet	4 (9)	5 (10)	15 (12)	28 (7)
6 years or less	20 (46)	25 (50)	42 (34)	134 (35)
Between 7 and 9 years	11 (26)	13 (26)	27 (21)	92 (24)
More than 9 years	8 (19)	7 (14)	41 (33)	130 (34)
***Occupation***				
Housewife	28 (65)	26 (52)	60 (48)	222 (58)
Informal employment	NA	18 (36)	48 (38)	130 (34)
Formal employment	NA	6 (12)	17 (14)	32 (8)
***Monthly family income***				
3 minimum wages or less	NA	39 (78)	89 (71)	243 (63)
More than 3 minimum wages	NA	10 (20)	24 (19)	79 (25)
Did not respond	NA	1 (2)	12 (10)	45 (12)
***Means of problem detection***				
Patient self discovery	38 (88)	43 (86)	101 (81)	297 (77)
Screening CBE or mammogram	5 (12)	7 (14)	24 (19)	87 (23)
***Final diagnosis***				
Benign disease	11 (26)	7 (14)	24 (19)	117 (30)
Cancer	31 (72)	43 (86)	98 (79)	259 (68)
Unconfirmed	1 (2)	0	3 (2)	8 (2)
***Cancer stage***				
Stage 0 & I	1 (3)	4 (8)	12 (12)	35 (14)
Stage II	12 (39)	22 (44)	37 (38)	86 (33)
Stage III	12 (39)	12 (24)	35 (36)	90 (34)
Stage IV	5 (16)	4 (8)	14 (14)	34 (13)
Unknown	1 (3)	8 (16)	0	14 (6)
***Delay intervals (days): median (IQR)***				
Total interval	272.50 (391.50)	255.00 (402.00)	261.00 (403.00)	234.50 (361.00)
Patient interval	30.00 (318.00)	23.00 (86.75)	14.00 (79.00)	11.00 (84.00)
Provider interval	136.50 (199.75)	135.00 (406.00)	167.00 (284.00)	141.00 (213.00)

^a^ Values are number (%) unless specified; *NA* Not Available, *CBE* Clinical Breast Exam, *IQR* Interquartile Range.

### Pre-test 2. Item reduction, reliability and date accuracy (Stage 3)

The second draft of the questionnaire, with 112 items, was applied to 50 patients with probable breast cancer on their arrival at INCAN between June 15 and July 8, 2009. Patient demographic and disease information is shown in Table 2. After the correlation matrix of variables and principal component analysis for each questionnaire dimension were done *(data not shown)*, the third draft of the questionnaire was reduced to 94 items.

#### Reliability

A test-retest was performed for 53 of the 66 final items measuring factors correlated with delay and the delay intervals. The *t*-test results comparing the means of the interval lengths are presented in Table 3. There were no significant mean differences between the two measurements.

**Table 3:** Test-retest of intervals

	**Mean (SD)**	**Corr.**	**Mean difference (95% CI)**
Total interval 1	305.63 (237.47)	0.72***	62.97 (−1.74,127.68)
Total interval 2	242.67 (222.44)		
Patient interval 1	83.63 (132.38)	0.70***	−27.37 (−81.37, 26.63)
Patient interval 2	111.00 (201.00)		
Provider interval 1	220.40 (227.56)	0.36*	76.57 (−5.67, 158.79)
Provider interval 2	143.83 (141.55)		

* p < 0.05, *** p < 0.001.

Questionnaire items showed quite good external consistency in general. Table 4 presents descriptive statistics for the scores obtained from each questionnaire dimension in Test 1 and Test 2. Correlations between pairs of measurements for individual items are not shown, but in summary, the results were as follows: 16 items had excellent consistency, with correlation coefficients equal to or greater than 0.75 (p<0.05); 32 items had moderate-to-good consistency, with correlation coefficients between 0.4 and 0.75 (p<0.05); and 18 had poor consistency, with coefficients less than 0.4, although as is argued in the discussion section, this finding could be an effect of the passage of time.

**Table 4:** Test-retest dimension scores

	**Mean (SD, range)**	
**Dimensions**	**Test 1**	**Test 2**
*Total questionnaire score*	30 (8.48, 12–44)	27 (8.65, 11–41)
1. Means of problem identification	1 (0.38, 0–1)	1 (0.41, 0–1)
2. First symptom identified by the patient	1 (0.51, 0–1)	0 (0.51, 0–1)
3. Patient initial interpretations of symptoms	4 (2.35, 1–8)	4 (2.31, 1–8)
4. Breast cancer symptoms present at arrival to the cancer hospital	2 (1.66, 0–6)	2 (1.52, 0–5)
5. Most worrisome symptom for the patient	0 (0.51, 0–1)	0 (0.49, 0–1)
6. Patient’s reason for seeking medical care	1 (0.44, 0–1)	1 (0.51, 0–1)
7. Patient’s perception of patient delay	2 (1.65, 0–3)	2 (1.21, 0–3)
8. Patient’s perceived reasons for patient delay	4 (2.45, 0–8)	3 (2.65, 0–9)
9. Patient’s perceived barriers to have caused provider delay	NA	NA
10. Health service utilization	6 (2.27, 1–10)	5 (2.31, 1–9)
11. Social network support for medical attention	5 (0.50, 4–5)	5 (0.51, 4–5)
12. Time between problem identification and talking to someone	0 (0.41, 0–1)	0 (0.42, 0–1)
13. Use of alternative medicine	1 (0.41, 0–1)	0 (0.18, 0–1)
14. Cancer-related knowledge	3 (1.11, 0–5)	3 (1.09, 1–5)
15. Cancer screening practices	2 (1.22, 0–5)	2 (1.43, 0–5)

*SD* Standard deviation, *NA* Not Available.

#### Best time for the interviews

After testing different moments for performing the interviews, we determined that the best moment for the interview to take place was before the patient was seen by the breast specialist for the first time after her arrival to INCAN. At this moment, patient cooperation and recall of dates and events prior to her arrival at INCAN were maximised and interference with institutional procedures was minimised. While 66.7% (20/30) were able to give a precise date for problem identification in the first interview, in the second interview (three months later), only 36.7% (11/30) were able to recall a precise date. Similarly, 83% (25/30) were able to remember the precise date of the first medical consultation in the first interview, and only 56.7% (17/30) were able to do so in the second interview.

### Pre-test 3. Internal consistency (Stage 4)

125/167 new patients that arrived for the first time at the Breast Tumors Department of INCAN between July 20th and October 5th of 2009, were interviewed (Table 2). The questionnaire dimensions showed good internal consistency in this sample, with Cronbach’s alphas in the range of 0.51 to 0.90 (data not shown).

### Final test. Item selection and validity assessment (Stage 5)

Five hundred and sixteen women who arrived at the Breast Clinic of INCAN between October 2009 and July 2010 were invited to participate in test 4. The sociodemographic characteristics of the 384 participants are summarised in Table 2.

**Figure 2:**
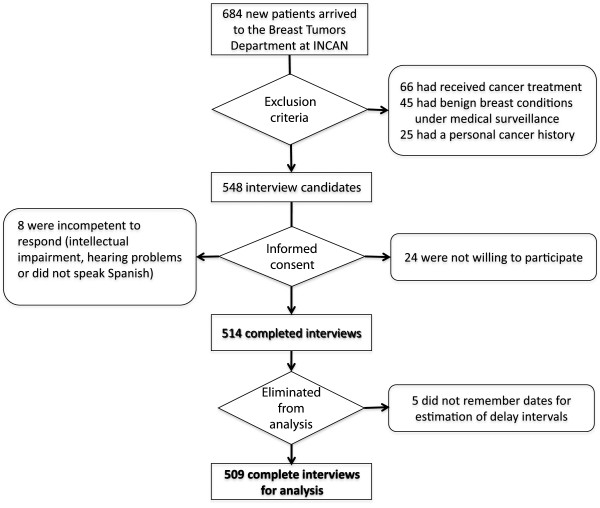
**Patients’ inclusion, exclusion and elimination criteria.** This diagram illustrates the process of invitation, exclusion, informed consent and elimination of study participants of the pretest 3 and test 4.

The following analyses were performed with patient samples from tests 3 and 4, for a total sample size of 509 patients. Figure 2 summarises the inclusion, exclusion and elimination criteria.

#### Final item selection

The majority of the final items that were selected had significantly high LR correlation coefficients and bivariate logistic regression ORs. Among the items with the highest associations (LR>10 and/or OR>2) with total delay were perceived medical errors (LR=26.27, p=0.000; OR=4.02, 95% CI: 2.26-7.14; p=0.000), difficulty missing work (LR=11.96, p=0.001; OR=3.39, 95% CI:1.59-7.23; p=0.002,), long waiting times for medical appointments (LR=11.96, p=0.001; OR=3.49, 95% CI:1.59-7.23; p=0.002,), having consulted more than two different health services prior to arrival at INCAN (LR=19.03, p=0.000; OR=2.78,, 95% CI:1.74-4.42; p=0.000), the patient’s reason for seeking medical help being worsening or persistence of symptoms rather than appearance (LR=13.92, p=0.000; OR=2.61,, 95% CI:1.57-4.33; p=0.000) and the patient taking longer than 30 days to talk to someone about her breast problem (LR=9.57, p=0.002; OR=2.28,, 95% CI:1.32-2.90; p=0.003). The majority of the items listed under the social network of support for medical attention were not significant. Nevertheless, they were kept because of their theoretical relevance.

#### Sensibility analysis

The instrument is comprehensible and replicable, but we strongly recommend that people who are to administer it receive training. The output scales have comprehensive and mutually exclusive response categories and allow discrimination of different features. The items have face validity, as they elicit the intended information, and they were well accepted and generally easily understood by both interviewers and interviewees.

Content validity was assured in the construction of the questionnaire by including items derived from the qualitative interviews and the literature and by having the appropriateness of included items reviewed by a multidisciplinary team.

The administration of the questionnaire may be time-consuming, taking in average 40 (SD=14.6) minutes. The instrument is usually more time-consuming to administer to older women and to those with no or incomplete primary school education, as these women have greater difficulty in remembering dates and understanding some questions. The instrument was, however, well accepted by patients, with only 4.4% (24/540) of those invited refusing to participate after the study was explained to them (Figure 2). Patient cooperation during the interview was good, and participants were usually highly motivated. After the interview, the majority of patients thanked the interviewer for listening to them.

The total score for the questionnaire ranged from 11 to 67 points, with a mean of 37 (SD = 11.52) points (n=509).

### Construct validity

#### Internal consistency

The instrument has fairly good internal consistency, with Cronbach’s alpha for each dimension in the range of 0.42 to 0.85 (Table 5). Although we tried to build a scale for the items related to the social network of support, as well as for those in the health service utilisation dimension, these items had very low Cronbach’s alpha values and were therefore left disaggregated.

**Table 5:** Internal consistency of questionnaire scales

**Questionnaire scales**	**Cronbach alphas**	**95% CI**
Symptom interpretation	0.77***	0.72-0.80
Symptoms at arrival to cancer hospital	0.59***	0.53-0.65
Reasons to delay seeking medical care	0.85***	0.82-0.87
Perceived barriers to reach cancer hospital	0.73***	0.69-0.77
Breast cancer knowledge	0.50***	0.43-0.56
Cancer screening practices	0.42***	0.33-0.49

*** p<0.001.

#### Convergent and divergent validity

The majority of items within the following dimensions showed moderate degrees of correlation with each other: *patient’s initial interpretation of symptoms* (r=0.52 – 0.72, *p*=0.000), *reasons for delay in initial seeking of care* (r= 0.21 – 0.64, *p*=0.000) and *perceived barriers in the provider interval* (r= 0.23 – 0.62, *p*=0.000). Items in the following dimensions correlated poorly (*r* ≤ 0.30): *symptoms present on arrival at the cancer hospital*, *social-network support for seeking medical care*, *health service utilisation*, *cancer-related knowledge* and *cancer-screening practices*. Items belonging to different dimensions in general were not correlated or were poorly correlated (*r* < 0.30), except for *perception of patient delay*, which was moderately correlated with most of the *perceived reasons for patient delay* and with *time between identification of the problem and the first time talking to someone about it* (r =0.35 – 0.74, *p*=0.000). Additionally, *perception of provider delay* was correlated with most of the *perceived barriers for provider delay* (r = 0.29 – 0.69, *p*=0.000).

#### Sensitivity and Specificity

The results derived from a ROC curve analysis are presented in Table 6. The questionnaire score predicted total, patient and provider delays with sensitivities of 74.68%, 66.67% and 81.17% and specificities of 48.81%, 77.70% and 20.24%, respectively. In general, it shows fair sensitivity and poor specificity, with higher specificity for patient delay than for provider delay.

**Table 6:** ROC Curve results of the total questionnaire score for the different delay intervals

	**Score cutpoint**	**Sensitivity (%)**	**Specificity (%)**	**LR+**	**AUC (95% CI)**
Total delay	40	74.68	48.81	1.46	0.62 (0.57-0.69)
Patient delay	55	66.67	77.80	3.00	0.72 (0.61-0.84)
Provider delay	40	81.17	20.24	1.02	0.51 (0.46-0.55)

*LR+* Likelihood Ratio Positive, *AUC* Area under the ROC Curve, *CI* Confidence Interval.

## Discussion

To the best of our knowledge, this is the first published report of the development and validation of a questionnaire for estimation of the breast cancer total, patient and provider intervals and the correlated factors with delays. Due to the association between delay and prognosis for breast cancer [32], it is important to quantify it and identify correlated factors. This is especially relevant in developing countries, where the majority of patients are diagnosed in advanced stages, as in Mexico [33].

The instrument was developed, modified and validated using standardised test-construction methods [28,34,40]. The results of the current study show that the instrument has good face validity, comprehensibility, patient acceptance, and content validity. It has acceptable internal consistency in most dimensions (considering the social nature of most of the items) and very good reliability for most items [34,41].

Although the two modules of the questionnaire were intertwined to facilitate its administration for this study, they would be easy to separate if someone wished to measure only the time intervals in another context. Nonetheless, we found that this intertwining of modules facilitated the questionnaire’s administration in our population, easing the flow of the interview.

Reliability was high for both *total* and *patient* intervals. In agreement with other studies, this shows that patients tend to recall the precise time when they first discovered their symptoms [12,31] or at least the month and year of this discovery [23]. Nevertheless, the *provider* interval did not show very good consistency. Because the test-retest measurements were taken with a 3-month separation, the variation in the estimation of this interval may be explained by recall bias. Apparently, memory was more affected in our participants in relation to the first medical consultation than in relation to the beginning of the problem. It is likely that the passage of time makes it harder for patients to recall dates and events that occurred prior to their admission, especially as medical consultations might blend with in-hospital consultations. This process could be even more challenging for patients who have already started chemotherapy (as was the case of most of our retest participants) because common secondary effects of chemotherapy include memory loss and difficulty concentrating [42]. These findings suggest that to minimise recall bias when assessing delay, the patients should be interviewed as early as possible, as has been suggested in previous studies [12,31].

Recall bias also seems to explain the poor external consistency scores of some other questionnaire items, including symptoms present when the patient first arrived at INCAN, the diagnosis offered by the first doctor consulted, the tests requested by this first doctor, and the time that passed from identification of the problem until the patient told someone about the problem. The remaining poor test-retest consistency scores could be explained by changes in these items over time. The first interview took place before the cancer diagnosis, while the second interview took place after treatment had begun. Items with poor consistency that could have changed over time include use of alternative medicine, knowing a person with cancer and knowledge of recommended breast cancer screening practices.

In regard to the length of the intervals reported in our findings, the median total interval was very prolonged for our study population (median: 234.5 days) and the main delays seem to be presenting within the provider interval (median: 151 days). This is similar to findings of studies that have been done in other Latin American countries. For example, another Mexican study that was done with a small sample of breast cancer patients (n=32) with public health insurance reported a mean *total interval* of 8.4 months [43]. A Colombian study of 1106 breast cancer patient files reported a median time from the first medical consultation to diagnosis confirmation (*diagnosis interval*) of 91 days [44] and a Brazilian study with 104 patients reported a median *diagnosis* interval of 6.5 months [45].

Another matter that we would like to briefly discuss is related to whether a self-complete tool could have provided a more objective way of administering the questionnaire. Such a tool could certainly reduce inter-observer variability and response bias [30]. It has been shown, however, that self-complete tools are difficult for undereducated populations (like our study population) to use [36,37]. This strategy would have most likely yielded a high rate of incomplete and inadequately filled-out questionnaires.

One limitation of our study is that external consistency measures are not available for some items because these items were identified as relevant only after the second pilot study. Because these items are “patient perceptions of barriers within the provider interval”, we hypothesise that they are likely to have reliabilities similar to those found for “perceptions of barriers within the patient interval” (moderate; between 0.4 and 0.75).

One more limitation was the impossibility of constructing scales for the social network of support for medical attention and health service utilisation. Another limitation was the lack of correlation between most of the social network items and the delay intervals. Nevertheless, we think that it is still premature to decide which of these items should be kept or discarded before the instrument is tested in a greater and more heterogeneous sample with higher levels of education, formal employment and higher socioeconomic status.

A disadvantage of the instrument is that its application is time-consuming, especially for older participants and those with low levels of education. On the other hand, it has high acceptability for patients, especially if it is administered when they first arrive at the hospital, before they see the breast specialist.

## Conclusions

On the basis of our analyses, we conclude that the Breast Cancer Delay Questionnaire is a valid, reliable and sensitive measure of the *total*, *patient* and *provider intervals* and the predisposing factors of delay in uninsured breast cancer patients treated at a public institution in Mexico. We think it could be useful in other settings, with similar populations, although it would have to be contextually adapted.

## Competing interests

The authors declare that they have no competing interests.

## Authors’ contributions

KUS conceived and designed the study, collected data, performed the analysis, interpreted the results and drafted the manuscript. IPB contributed in the study design, directed and supervised data analysis, contributed in the interpretation of results and critically reviewed the manuscript for important intellectual content. CIC contributed in the study design and critically reviewed the manuscript for important intellectual content. All authors read and approved the final manuscript.

## Authors’ information

KUS is a medical doctor with a Master’s Degree and a PhD in Health Systems. This research was part of her PhD research thesis. IPB is a medical doctor and archaeologist with a Master’s Degree in Clinical Epidemiology and a PhD in Medical Anthropology. CIC is a sociologist with a Master’s Degree in Medical Sociology and a PhD in Health Systems.

## Pre-publication history

The pre-publication history for this paper can be accessed here:

http://www.biomedcentral.com/1471-2407/12/626/prepub

## Supplementary Material

Additional file 1 The breast cancer delay questionnaire. Click here for file
